# Supplementary material for: Evaluating the accuracy of a nutritional screening tool for patients with digestive system tumors: A hierarchical Bayesian latent class meta-analysis
Source: PLoS One. 2024 Dec 20;19(12):e0316070. doi: 10.1371/journal.pone.0316070 (PMC11661584; doi:10.1371/journal.pone.0316070)
Supplement: S1 File — This file provides a detailed table documenting the inclusion and exclusion of studies during the systematic review, along with the reasons for exclusion. (DOC) [file pone.0316070.s001.doc]

**Literature Screening and Exclusion Reasons**

| Study ID | Author (Year) | Included/Excluded | Reason for Exclusion |
| --- | --- | --- | --- |
| 1 | Wang ZB(2021) | Included | N/A |
| 2 | Guo T(2015) | Included | N/A |
| 3 | Yang D(2022) | Excluded | Data incomplete |
| 4 | Li M(2017) | Excluded | Not a diagnostic study |
| 5 | Li G(2018) | Excluded | Suspected duplicate publication |
| 6 | Jin T(2010) | Included | N/A |
| 7 | Hu H(2018) | Excluded | Data incomplete |
| 8 | Wu JC(2023) | Excluded | Data incomplete |
| 9 | Huang D(2017) | Excluded | Suspected duplicate publication |
| 10 | Wang C(2021) | Included | N/A |
| 11 | Huang D(2018) | Included | N/A |
| 12 | Wang L(2024) | Excluded | Data incomplete |
| 13 | Liu YQ(2017) | Included | N/A |
| 14 | Huang T(2018) | Excluded | Not a diagnostic study |
| 15 | Huang B(2014) | Excluded | Data incomplete |
| 16 | Ji Y(2018) | Excluded | Not a diagnostic study |
| 17 | Li X(2018) | Included | N/A |
| 18 | Chen B(2021) | Excluded | Data incomplete |
| 19 | Xu X(2017) | Excluded | Data incomplete |
| 20 | Zhang ZP(2020) | Excluded | Not a diagnostic study |
| 21 | Qiao K(2009) | Excluded | Data incomplete |
| 22 | Gui L(2016) | Excluded | Data incomplete |
| 23 | Ji H(2023) | Included | N/A |
| 24 | Yang D(2018) | Excluded | Suspected duplicate publication |
| 25 | Guo W(2010) | Included | N/A |
| 26 | Li L(2024) | Excluded | Not a diagnostic study |
| 27 | Han D(2015) | Excluded | Data incomplete |
| 28 | Liu P(2013) | Included | N/A |
| 29 | Li J(2024) | Excluded | Not a diagnostic study |
| 30 | Zhou Y(2017) | Included | N/A |
| 31 | Zhou W(2015) | Included | N/A |
| 32 | Lin L(2018) | Included | N/A |
| 33 | Fang L(2020) | Excluded | Data incomplete |
| 34 | Xiao Y(2016) | Excluded | Not a diagnostic study |
| 35 | Liang H(2020) | Included | N/A |
| 36 | Yu L(2022) | Excluded | Not a diagnostic study |
| 37 | Liu Y(2018) | Excluded | Not a diagnostic study |
| 38 | Fu L(2017) | Excluded | Data incomplete |
| 39 | Zhu Y(2021) | Included | N/A |
| 40 | Song P(2020) | Excluded | Data incomplete |
| 41 | Shi Y(2019) | Included | N/A |
| 42 | Yin H(2021) | Excluded | Data incomplete |
| 43 | Li X(2016) | Excluded | Data incomplete |
| 44 | Li X(2015) | Included | N/A |
| 45 | Putting M(2023) | Excluded | Data incomplete |
| 46 | Xie B(2022) | Included | N/A |
| 47 | Si Y(2021) | Excluded | Data incomplete |
| 48 | Tang S(2021) | Excluded | Not a diagnostic study |
| 49 | Weng M(2021) | Excluded | Not a diagnostic study |
| 50 | Liang X(2013) | Excluded | Data incomplete |
| 51 | Lin X(2023) | Excluded | Not a diagnostic study |
| 52 | Li G(2019) | Included | N/A |
| 53 | Zhuo J(2022) | Excluded | Not a diagnostic study |
| 54 | Ruan X(2022) | Included | N/A |
| 55 | Huang DD(2020) | Excluded | Not a diagnostic study |
| 56 | Heckler M(2021) | Excluded | Data incomplete |
| 57 | Zheng X(2024) | Included | N/A |
| 58 | Pereira Borges N(2009) | Excluded | Not a diagnostic study |
| 59 | Tan S(2022) | Included | N/A |
| 60 | Wakahara T(2007) | Excluded | Data incomplete |
| 61 | Tan S(2024) | Included | N/A |
| 62 | Chen X(2020) | Excluded | Not a diagnostic study |
| 63 | Huang S(2021) | Excluded | Data incomplete |
| 64 | Yildirim R(2020) | Included | N/A |
| 65 | Lee JY(2022) | Excluded | Not a diagnostic study |
| 66 | Sun W(2022) | Excluded | Data incomplete |
| 67 | Zhang L(2022) | Excluded | Not a diagnostic study |
| 68 | Durán Poveda M(2023) | Excluded | Not a diagnostic study |
| 69 | Wang H(2022) | Excluded | Not a diagnostic study |
| 70 | Hsueh SW(2020) | Excluded | Data incomplete |
| 71 | Lu JL(2022) | Excluded | Not a diagnostic study |
| 72 | Xie B(2022) | Included | N/A |
| 73 | Abe Vicente M(2013) | Included | N/A |
| 74 | Ryu SW(2010) | Included | N/A |
| 75 | Yang D(2020) | Included | N/A |
| 76 | Tu MY(2012) | Included | N/A |
| 77 | Chi J(2017) | Included | N/A |
| 78 | Faramarzi E(2013) | Included | N/A |
| 79 | Ye XJ(2018) | Included | N/A |
| 80 | Zhang Q(2022) | Included | N/A |

**Explanation:**

This table provides a comprehensive overview of the literature screening process for the systematic review. All studies identified during the initial database search are listed with their inclusion or exclusion status and the reasons for exclusion, if applicable.

1.Study ID: A unique identifier assigned to each study for easy reference.

2.Author (Year): The primary author and publication year of each study.

3.Included/Excluded: Indicates whether the study was included in the final analysis.

4.Reason for Exclusion: For studies excluded from the analysis, this column details the specific reasons, such as "Data incomplete," "Not a diagnostic study," or "Suspected duplicate publication."

This table ensures transparency in the study selection process and helps verify the criteria used for inclusion and exclusion.

**References**

1. wang ZB, Li X, Liu HZ, et al. Comparison of preoperative nutritional status and different nutritional screening assessment methods in 63 male esophageal cancer patients. Journal of Weifang Medical College. 2021;43(06):459-463.
2. Guo T. Investigation and research on the nutritional status of 100 cases of colorectal/rectal cancer patients [Dissertation]. Hebei Medical University; 2015.
3. Yang D, Yang YG, Liu W, Li JX. Survey on nutritional risk, malnutrition and perioperative nutritional support treatment in 118 esophageal cancer patients. Chinese Science and Technology Journal Database (Citation Edition) Medicine and Health. 2022;(4):238-242.
4. Li M, Qi F, Ju K, et al. Analysis of preoperative MNA-SF and NRS2002 nutritional screening results and prognosis of 242 cases of elderly colorectal cancer. Hebei Medicine. 2017;23(10):1623-1628.
5. Li G, Wu X, Huang D, et al. Application effect of ERAS in patients undergoing radical gastric cancer surgery and analysis of PG-SGA and NRS2002 assessment. China Practical Medicine. 2018;13(14):1-3.
6. Jin T. Research on the application of ESPEN Nutritional Risk Screening (NRS2002) in preoperative gastric cancer patients [Dissertation]. Chongqing Medical University; 2010.
7. Hu H, Cheng Y, Cao H, Chen X, Kuang B, Gong P. Comparison of the application of two scales, MNA-SF and NRS2002, to assess the nutritional status of elderly gastrointestinal tumor inpatients. Medical Clinical Research. 2018;35(1):97-99.
8. Wu JC, Zeng JD, Tsang JQ, Huang W, Xiao X, Sheng Z. Comparison of the application of MSTC and NRS2002 in patients with gastrointestinal malignant tumors. Jiangxi Medicine. 2023;58(8):944-947.
9. Huang D, Wu X, Chen J, et al. The roles of NRS2002, PG-SGA, BMI, and TF in nutritional screening and assessment of gastric cancer. Electronic Journal of Oncology Metabolism and Nutrition. 2017;4(3):317-321.
10. Wang C. Study on the correlation between NRS2002, PG-SGA and clinical nutritional indexes and pathological staging of gastric cancer patients [Dissertation]. Guangxi Medical University; 2021.
11. Huang D. Application of NRS2002, PG-SGA and serum biochemical indexes in perioperative and postoperative complications of gastric cancer patients [Dissertation]. Guangxi Medical University; 2018.
12. Wang L, Yuan YF, Liu X. Application of NRS-2002 and PG-SGA in nutritional screening and assessment of patients with middle and advanced tumors. Hainan Med. 2024;35(2):210-214.
13. Liu YQ. Comparison of NRS-2002 and PG-SGA in chemotherapy patients with advanced primary digestive tumors [Dissertation]. Shanxi Medical University; 2017.
14. Huang T, Zhong J, Zhang Q. The value of nutritional risk screening 2002 in guiding perioperative nutritional support in colorectal cancer patients. Modern Digestive and Interventional Diagnosis and Treatment. 2018;23(6):765-767.
15. Huang B, Wang Q. Research on nutritional risk screening and nutritional support status of colorectal cancer patients. Chinese Family Medicine. 2014;17(6):656-658.
16. Ji Y, Shen T, Ma B, Ye X, Huang D, Zhong C, Jia Z, Yu Z. Study on the predictive value of three nutritional screening tools for complications after laparoscopic colorectal cancer resection. Chinese Journal of Practical Surgery. 2018;38(3):301-305.
17. Li X, Qu Q, He W. Application of modified version of patient subjective holistic assessment scale in nutritional assessment of gastric cancer patients undergoing postoperative concurrent chemotherapy. Chinese Journal of Modern Nursing. 2018;24(19):2261-2266.
18. Chen B, Huang S, Xu X, Chen C, Zheng X. Study on the effect of NRS-2002 for nutritional risk screening in hospitalized patients with esophageal cancer. International Medical and Health Herald. 2021;27(16):2460-2463.
19. Xu X. Comparison of NRS2002 and MNA-SF nutritional screening tools in pancreaticoduodenectomy [Dissertation]. Xinjiang Medical University; 2017.
20. Zhang ZP. Comparison of the value of three nutritional screening tools for predicting complications after radical gastric cancer surgery [Dissertation]. Qingdao University; 2020.
21. Qiao K, Hu K, Ou Z, et al. Comparison of different screening tools for nutritional screening of esophageal cancer patients. Journal of Digestive Oncology (Electronic Edition). 2009;1(01):59-61.
22. Gui L. Comparative study of different screening tools for nutritional screening of esophageal cancer patients. Hebei Medicine. 2016;22(12):2002-2005.
23. Ji H, Hou J, Meng Y, et al. Analysis of sensitivity, specificity and precision of different nutritional risk screening tools for assessing nutritional status in hepatocellular carcinoma patients. Modern Digestive and Interventional Diagnosis and Treatment. 2023;28(06):758-761.
24. Yang D, Zheng Z, Zhao Y, et al. Comparison of different scoring systems for preoperative assessment of nutritional status in patients with gastric cancer with pyloric obstruction. Tianjin Medicine. 2018;46(08):865-868.
25. Guo W, Chen T, Au G, et al. Comparison of different nutritional scoring systems for preoperative nutritional risk assessment in gastric cancer patients. Chinese Journal of Gastrointestinal Surgery. 2010;9(2):140-142.
26. Li L, Jiang W, Li Y, Liu D, Zhang Y, Gao C. The value of prognostic nutrition index in the prognostic assessment of patients after radical gastric cancer surgery. Zhejiang Med. 2024;46(9):913-919.
27. Han D, He J, Ding C, He G. Comparison of the assessment value of different nutritional evaluation methods for postoperative complications in cholangiocarcinoma patients. Chinese Journal of Modern Medicine. 2015;25(8):88-92.
28. Liu P. Study on the applicability of different nutritional evaluation methods to primary liver cancer patients [Dissertation]. Tianjin Medical University; 2013.
29. Li J, Wang M, Wu C, et al. Predictive value of different nutritional indicators for postoperative debility in elderly patients with gastrointestinal tumors. Practical Geriatrics. 2024;38(02):157-161.
30. Zhou Y. Follow-up study on nutritional status of gastric cancer patients in different treatment stages [Dissertation]. Southeast University; 2017.
31. Zhou W, Xu Q, Yan K, et al. Evaluation of the applicability of a modified version of the Patient Self-Assessed Subjective Overall Nutritional Assessment Scale in patients undergoing chemotherapy for gastrointestinal tumors. Journal of Nursing. 2015;30(01):20-22.
32. Lin L, Zhang J, Xie F, et al. Comparison of patient comprehensive subjective nutritional assessment and nutritional risk screening 2002 in nutritional assessment of digestive system tumor patients. China Food and Nutrition. 2018;24(03):78-82.
33. Fang L, Xiong X, Fang L, et al. Exploring the nutritional assessment and prognostic value of four nutritional screening tools for patients with hepatocellular carcinoma based on ESPEN criteria. Journal of Practical Medicine. 2020;36(20):2792-2796, 2801.
34. Xiao Y, He Y. Analysis of the relationship between nutritional risk scores and corresponding nutritional support and postoperative complications in various stages of colorectal cancer. Medical Clinical Research. 2016;33(6):1236-1238.
35. Liang H, Wu W, Jia S, et al. Survey on nutritional status and pharmacists' nutritional intervention in patients hospitalized with colorectal cancer. Practical Drugs and Clinics. 2020;23(7):632-636.
36. Yu L, Zhao J, Jiang G. Selection and application of nutritional assessment tools for elderly patients with esophageal cancer. International Journal of Geriatrics. 2022;43(4):434-437.
37. Liu Y, Peng Y, Zhou J, Gao Z. Investigation and analysis of preoperative nutritional status and nutritional support in elderly gastrointestinal tumor patients. Journal of PLA Nursing. 2018;35(7):29-31.
38. Fu L. Predictive value of two preoperative nutritional evaluation methods for postoperative complications in patients with gastrointestinal malignant tumors. Journal of Clinical and Experimental Medicine. 2017;16(7):708-711.
39. Zhu Y, Wu Y, Wang J, Chen T, Yang Y. Comparison of two malnutrition risk screening tools in hospitalized patients with primary liver cancer. Medical Review. 2021;27(8):1616-1620.
40. Song P, Mao L, Bian X, et al. Comparison of two nutritional risk screening methods in the perioperative period of hepatic resection for hepatocellular carcinoma under accelerated rehabilitation model. Chinese Electronic Journal of Liver Surgery. 2020;9(03):221-226.
41. Shi Y, Bian X. Comparison of NRS2002 and PG-SGA preoperative assessment and screening for nutritional risk in gastric cancer patients. Journal of Practical Medicine. 2019;36(06):501-503.
42. Yin H, Deng M, Wang Y, et al. Comparison of three nutritional screening tools for the evaluation of malnutrition in patients with esophageal cancer. Tumor Prevention and Treatment. 2021;34(02):127-132.
43. Li X. Comparative study of nutritional screening of esophageal cancer patients with different screening tools. Journal of Practical Clinical Medicine. 2016;20(20):154-155.
44. Qiao X. Clinical research on the assessment of nutritional status and quality of life in patients with progressive gastric cancer [Dissertation]. Hebei Medical University; 2015.
45. Putting M. Analysis of the value of preoperative NRS2002 and PNI nutritional screening methods in the prediction of postoperative complications in gastrointestinal tumors [Dissertation]. Anhui Medical University; 2023.
46. Xie B. A study on the applicability of three different nutritional risk screening scales in colorectal cancer surgery patients [Dissertation]. China Medical University; 2022.
47. Si Y, Ma T. Application of preoperative nutritional risk screening and nutritional support intervention in patients with gastrointestinal malignant tumors. Clinical Research. 2021;29(10):159-161.
48. Tang S, Wang H, Xia C, et al. Survey on nutritional status of patients treated with chemotherapy for malignant tumors of the digestive tract and analysis of influencing factors. Parenteral and Enteral Nutrition. 2021;28(01):35-40.
49. Weng M, Dai Z, Gan C, et al. Correlation analysis between nutritional status and quality of life of inpatients with malignant tumors of the digestive system. Parenteral and Enteral Nutrition. 2021;28(06):347-351.
50. Liang X, Xu G. Nutritional risk screening 2002 and patient overall subjective scoring method in preoperative patients with esophageal cancer. Chinese Journal of Practical Nursing. 2013;29(22):27-29.
51. Lin X, Dong C, Tong LL. Application of total nutrition management based on malnutrition risk screening tool 2002 in patients operated for gastrointestinal malignant tumors. Medical Equipment. 2023;36(21):25-29.
52. Li G. Study on the relevance of nutrition-related blood biochemical indexes and anthropometric measurements in nutritional screening and assessment of gastric cancer patients [Dissertation]. Guangxi Medical University; 2019.
53. Zhuo J, Li Z, Han T. The value of prognostic nutrition index and phase angle in the diagnosis of malnutrition in colorectal tumor patients. Parenteral and Enteral Nutrition. 2022;29(03):129-134.
54. Ruan X, Wang X, Zhang Q, et al. The performance of three nutritional tools varied in colorectal cancer patients: a retrospective analysis. J Clin Epidemiol. 2022;149:12-22.
55. Huang DD, Cai HY, Chen XY, et al. Value of Sarcopenia defined by the new EWGSOP2 consensus for the prediction of postoperative complications and long-term survival after radical gastrectomy for gastric cancer: A comparison with four common nutritional screening tools. J Cancer. 2020;11(19):5852-5860.
56. Heckler M, Klaiber U, Hüttner FJ, et al. Prospective trial to evaluate the prognostic value of different nutritional assessment scores for survival in pancreatic ductal adenocarcinoma (NURIMAS Pancreas SURVIVAL). J Cachexia Sarcopenia Muscle. 2021;12(6):1940-1947.
57. Zheng X, Ruan X, Wang X, et al. Bayesian diagnostic test evaluation and true prevalence estimation of malnutrition in gastric cancer patients. Clin Nutr ESPEN. 2024;59:436-443.
58. Pereira Borges N, D'Alegria Silva B, Cohen C, et al. Comparison of the nutritional diagnosis, obtained through different methods and indicators, in patients with cancer. Nutr Hosp. 2009;24(1):51-5.
59. Tan S, Wang J, Zhou F, et al. Validation of GLIM malnutrition criteria in cancer patients undergoing major abdominal surgery: A large-scale prospective study. Clin Nutr. 2022;41(3):599-609.
60. Wakahara T, Shiraki M, Murase K, et al. Nutritional screening with Subjective Global Assessment predicts hospital stay in patients with digestive diseases. Nutrition. 2007;23(9):634-639.
61. Tan S, Jiang J, Qiu L, Liang Y, Meng J, Tan N, Xiang B. Prevalence of malnutrition in patients with hepatocellular carcinoma: A comparative study of GLIM criteria, NRS2002, and PG-SGA, and identification of independent risk factors. Nutr Cancer. 2024;76(4):335-344.
62. Chen X, Zhang X, Ma B, et al. A comparison of four common malnutrition risk screening tools for detecting cachexia in patients with curable gastric cancer. Nutrition. 2020;70:110498.
63. Huang S, Wang S, Xie Y, et al. Application of NRS2002 in preoperative nutritional screening for patients with liver cancer. J Oncol. 2021;2021:8943353.
64. Yildirim R, Candaş Altınbaş B, Usta M, et al. Comparison of nutritional screening tools in patients undergoing surgery for gastric cancer. Haseki Tip Bulteni-Medical Bulletin of Haseki. 2020;58(2).
65. Lee JY, Oh EG, Hyung WJ, Kim HI. Translation and validation of the patient-generated subjective global assessment against the mini-nutritional assessment for patients with gastric cancer. Asia Pac J Oncol Nurs. 2022;10(1):100148.
66. Sun W, Li G, Zhang J, et al. The role of nutritional assessment for predicting radiotherapy-induced adverse events in patients with gastric cancer. Br J Radiol. 2022;95(1130):20201004.
67. Zhang L, Wang S, Gao X, et al. Poor pre-operative nutritional status is a risk factor of post-operative infections in patients with gastrointestinal cancer: A multicenter prospective cohort study. Front Nutr. 2022;9:850063.
68. Durán Poveda M, Suárez-de-la-Rica A, Cancer Minchot E, et al. The prevalence and impact of nutritional risk and malnutrition in gastrointestinal surgical oncology patients: A prospective, observational, multicenter, and exploratory study. Nutrients. 2023;15(14):3283.
69. Wang H, Wang T, Huang C, et al. Nutritional status and related factors in patients with gastric cancer after gastrectomy: A cross-sectional study. Nutrients. 2022;14(13):2634.
70. Hsueh SW, Liu KH, Hung CY, et al. Predicting postoperative events in patients with gastric cancer: A comparison of five nutrition assessment tools. In Vivo. 2020;34(5):2803-2809.
71. Lu JL, Xu Q, Zhu SQ, et al. Comparison of five sarcopenia screening tools in preoperative patients with gastric cancer using the diagnostic criteria of the European Working Group on Sarcopenia in Older People 2. Nutrition. 2022;95:111553.
72. Xie B, Sun Y, Sun J, et al. Applicability of five nutritional screening tools in Chinese patients undergoing colorectal cancer surgery: A cross-sectional study. BMJ Open. 2022;12(5)
73. Abe Vicente M, Barão K, Silva TD, Forones NM. What are the most effective methods for assessment of nutritional status in outpatients with gastric and colorectal cancer? Nutr Hosp. 2013;28(3):585-591.
74. Ryu SW, Kim IH. Comparison of different nutritional assessments in detecting malnutrition among gastric cancer patients. World J Gastroenterol. 2010;16(26):3310-3317.
75. Yang D, Zheng Z, Zhao Y, et al. Patient-generated subjective global assessment versus nutritional risk screening 2002 for gastric cancer in Chinese patients. Future Oncol. 2020;16(3):4475-4483.
76. Tu MY, Chien TW, Chou MT. Using a nutritional screening tool to evaluate the nutritional status of patients with colorectal cancer. Nutr Cancer. 2012;64(2):323-330.
77. Chi J, Yin S, Zhu Y, et al. A comparison of the nutritional risk screening 2002 tool with the subjective global assessment tool to detect nutritional status in Chinese patients undergoing surgery with gastrointestinal cancer. Gastroenterol Nurs. 2017;40(1):19-25.
78. Faramarzi E, Mahdavi R, Mohammad-Zadeh M, Nasirimotlagh B. Validation of nutritional risk index method against patient-generated subjective global assessment in screening malnutrition in colorectal cancer patients. Chin J Cancer Res. 2013;25(5):544-548.
79. Ye XJ, Ji YB, Ma BW, et al. Comparison of three common nutritional screening tools with the new European Society for Clinical Nutrition and Metabolism (ESPEN) criteria for malnutrition among patients with geriatric gastrointestinal cancer: A prospective study in China. BMJ Open. 2018;8(4)
80. Zhang Q, Yu S, Li Q, et al. Preoperative nutritional status in elderly inpatients with gastrointestinal cancer and its linear association with frailty. Nutr Cancer. 2022;74(4):1376-1387.
