# Supplementary material for: Evaluating the accuracy of a nutritional screening tool for patients with digestive system tumors: A hierarchical Bayesian latent class meta-analysis
Source: PLoS One. 2024 Dec 20;19(12):e0316070. doi: 10.1371/journal.pone.0316070 (PMC11661584; doi:10.1371/journal.pone.0316070)
Supplement: S2 File — This file contains the data extracted from the included studies, including study characteristics, diagnostic performance metrics, and other relevant details used in the meta-analyses. (DOC) [file pone.0316070.s002.doc]

**Data Extracted from Included Studies**

| Author | Year | Data Extractor(s) | Date of Extraction | sample size | reference standard | Index test | TP | FP | FN | TN | Inclusion Confirmed |
| --- | --- | --- | --- | --- | --- | --- | --- | --- | --- | --- | --- |
| Zibing Wang | 2021 | Yang Menghao and Xiao Na | 15-Mar-24 | 63 | PG-SGA（A＋B/C) | NRS-2002≥3 | 18 | 17 | 2 | 26 | Y |
| Ting Guo | 2015 | Yang Menghao and Xiao Na | 15-Mar-24 | 100 | PG-SGA（A＋B/C) | NRS-2002≥3 | 14 | 29 | 3 | 54 | Y |
| Changli Wang | 2021 | Yang Menghao and Xiao Na | 15-Mar-24 | 248 | PG-SGA（A＋B/C) | NRS-2002≥3 | 105 | 48 | 9 | 86 | Y |
| Daolai Huang | 2018 | Yang Menghao and Xiao Na | 18-Mar-24 | 181 | PG-SGA（A＋B/C) | NRS-2002≥3 | 55 | 25 | 21 | 80 | Y |
| Yuqiang Liu | 2017 | Yang Menghao and Xiao Na | 18-Mar-24 | 99 | PG-SGA（A/B＋C） | NRS-2002≥3 | 35 | 2 | 15 | 47 | Y |
| Weiping Guo | 2010 | Yang Menghao and Xiao Na | 18-Mar-24 | 314 | SGA（A/B＋C） | MUST | 125 | 42 | 46 | 101 | Y |
|  |  |  |  |  |  | NRS-2002≥3 | 148 | 37 | 23 | 106 |  |
| Ping Liu | 2013 | Yang Menghao and Xiao Na | 18-Mar-24 | 80 | SGA（A/B＋C） | MNA＜24 | 18 | 18 | 0 | 44 | Y |
|  |  |  |  |  |  | NRS-2002≥3 | 17 | 10 | 1 | 52 |  |
| Yu Zhou | 2017 | Yang Menghao and Xiao Na | 19-Mar-24 | 196 | PG-SGA（A/B＋C） | NRS-2002≥3 | 71 | 3 | 87 | 35 | Y |
| Xiaojing Li | 2018 | Yang Menghao and Xiao Na | 19-Mar-24 | 103 | PG-SGA（≥4） | abPG-SGA | 51 | 9 | 7 | 36 | Y |
|  |  |  |  |  |  | NRS-2002≥3 | 35 | 21 | 23 | 24 |  |
|  |  |  |  |  |  | abPG-SGA | 60 | 6 | 8 | 29 |  |
|  |  |  |  |  |  | NRS-2002≥3 | 45 | 13 | 23 | 22 |  |
|  |  |  |  |  |  | abPG-SGA | 72 | 2 | 4 | 25 |  |
|  |  |  |  |  |  | NRS-2002≥3 | 57 | 8 | 19 | 19 |  |
| Wan Zhou | 2015 | Yang Menghao and Xiao Na | 20-Mar-24 | 150 | PG-SGA（≥4） | abPG-SGA | 75 | 1 | 6 | 68 | Y |
|  |  |  |  |  |  | NRS-2002≥3 | 69 | 15 | 12 | 54 |  |
| Hai Liang | 2020 | Yang Menghao and Xiao Na | 20-Mar-24 | 392 | PG-SGA（≥4） | NRS-2002≥3 | 196 | 12 | 96 | 88 | Y |
| Yage Zhu | 2021 | Yang Menghao and Xiao Na | 20-Mar-24 | 115 | SGA（A/B＋C） | NRS-2002≥3 | 65 | 2 | 18 | 30 | Y |
| Guibin Li | 2019 | Yang Menghao and Xiao Na | 20-Mar-24 | 187 | PG-SGA（≥4） | NRS-2002≥3 | 82 | 4 | 52 | 49 | Y |
| Juntao Chi | 2017 | Yang Menghao and Xiao Na | 21-Mar-24 | 280 | SGA（A/B＋C） | NRS-2002≥3 | 89 | 60 | 6 | 125 | Y |
| Shanjun Tan | 2022 | Yang Menghao and Xiao Na | 21-Mar-24 | 706 | SGA（A/B＋C） | NRS-2002≥3 | 108 | 13 | 177 | 408 | Y |
|  |  |  |  |  |  | MNA-SF≤11 | 205 | 72 | 80 | 349 |  |
|  |  |  |  |  |  | MUST | 182 | 46 | 103 | 375 |  |
| Elnaz Faramarzi | 2012 | Yang Menghao and Xiao Na | 21-Mar-24 | 52 | PG-SGA（A/B＋C） | NRI | 18 | 10 | 9 | 15 | Y |
| Mei-Yu Tu | 2012 | Yang Menghao and Xiao Na | 21-Mar-24 | 45 | SGA（A/B＋C） | NRI | 15 | 9 | 1 | 20 | Y |
|  |  |  |  |  |  | MUST | 15 | 5 | 1 | 24 |  |
|  |  |  |  |  | PA≤20mg/dL | MUST | 12 | 8 | 9 | 16 |  |
|  |  |  |  |  |  | NRI | 17 | 7 | 4 | 17 |  |
|  |  |  |  |  |  | SGA | 13 | 3 | 8 | 21 |  |
| Seung Wan Ryu | 2010 | Yang Menghao and Xiao Na | 22-Mar-24 | 80 | SGA（A/B＋C） | NRS-2002≥3 | 24 | 11 | 1 | 44 | Y |
|  |  |  |  |  |  | NRI | 10 | 15 | 15 | 40 |  |
| Bingxin Xie | 2022 | Yang Menghao and Xiao Na | 22-Mar-24 | 301 | SGA（A/B＋C） | NRS-2002≥3 | 99 | 36 | 32 | 134 | Y |
|  |  |  |  |  |  | MNA-SF≤11 | 97 | 29 | 34 | 141 |  |
|  |  |  |  |  |  | MUST | 96 | 41 | 35 | 129 |  |
|  |  |  |  |  |  | MST | 108 | 45 | 23 | 125 |  |
|  |  |  |  |  |  | NRI | 79 | 58 | 52 | 112 |  |
| Mariana Abe Vicente | 2013 | Yang Menghao and Xiao Na | 22-Mar-24 | 75 | PG-SGA（A/B＋C） | NRI | 34 | 9 | 16 | 16 | Y |
|  |  |  |  |  |  | MST | 26 | 4 | 24 | 21 |  |
|  |  |  |  |  |  | MUST | 36 | 13 | 14 | 12 |  |
|  |  |  |  | 62 | PG-SGA（A/B＋C) | NRI | 7 | 8 | 6 | 41 |  |
|  |  |  |  |  |  | MST | 8 | 4 | 5 | 45 |  |
|  |  |  |  |  |  | MUST | 11 | 13 | 2 | 36 |  |
| Taobo Jin | 2010 | Yang Menghao and Xiao Na | 22-Mar-24 | 56 | ALB<35g/L | NRS-2002≥3 | 13 | 6 | 3 | 34 | Y |
|  |  |  |  |  |  | NRI | 16 | 12 | 0 | 28 |  |
|  |  |  |  |  |  | MNA-SF≤11 | 12 | 26 | 4 | 14 |  |
| Yingying Shi | 2019 | Yang Menghao and Xiao Na | 25-Mar-24 | 168 | ALB≤30g/L | NNRS-2002≥3 | 5 | 77 | 0 | 86 | Y |
|  |  |  |  |  |  | PG-SGA | 5 | 124 | 0 | 39 |  |
| Hong Ji | 2023 | Yang Menghao and Xiao Na | 25-Mar-24 | 76 | the ESPEN diagnostic criteria | NRS-2002≥3 | 51 | 2 | 18 | 5 | Y |
|  |  |  |  |  |  | PG-SGA | 64 | 1 | 5 | 6 |  |
| Li Lin | 2018 | Yang Menghao and Xiao Na | 25-Mar-24 | 680 | ALB≤30g/L | NRS-2002≥3 | 192 | 62 | 44 | 376 | Y |
|  |  |  |  |  |  | PG-SGA | 205 | 41 | 37 | 397 |  |
| Xi Qiao | 2015 | Yang Menghao and Xiao Na | 26-Mar-24 | 457 | BMI＜18.5 kg/m2/ALB＜35 g/L | NRS-2002≥3 | 104 | 137 | 10 | 216 | Y |
|  |  |  |  |  |  | PG-SGA | 108 | 248 | 6 | 105 |  |
| Bingxin Xie | 2022 | Yang Menghao and Xiao Na | 26-Mar-24 | 280 | BMI＜18.5 kg/m2/ALB＜35 g/L | NRS-2002≥3 | 35 | 81 | 12 | 152 | Y |
|  |  |  |  |  |  | MUST | 30 | 84 | 17 | 149 |  |
|  |  |  |  |  |  | MNA-SF≤11 | 34 | 95 | 13 | 138 |  |
| Xite Zheng | 2024 | Yang Menghao and Xiao Na | 26-Mar-24 | 1308 | the fitting Bayesian LCM analysis | NRS-2002≥3 | 646 | 38 | 348 | 276 | Y |
|  |  |  |  |  |  | PG-SGA | 954 | 41 | 40 | 273 |  |
| Shengqiang Tan | 2024 | Yang Menghao and Xiao Na | 26-Mar-24 | 207 | the GLIM diagnostic criteria | NRS-2002≥3 | 57 | 6 | 32 | 112 | Y |
|  |  |  |  |  |  | PG-SGA | 84 | 29 | 5 | 89 |  |
| Xiaoli Ruan | 2022 | Yang Menghao and Xiao Na | 26-Mar-24 | 1358 | the fitting Bayesian LCM analysis | NRS-2002≥3 | 486 | 60 | 274 | 538 | Y |
|  |  |  |  |  |  | PG-SGA | 730 | 108 | 30 | 490 |  |
| Reyyan Yıldırım | 2020 | Yang Menghao and Xiao Na | 27-Mar-24 | 140 | the ESPEN diagnostic criteria | NRS-2002≥3 | 23 | 53 | 6 | 58 | Y |
|  |  |  |  |  |  | MUST | 25 | 34 | 4 | 77 |  |
|  |  |  |  |  |  | SGA | 19 | 40 | 10 | 71 |  |
|  |  |  |  |  |  | MNA-SF≤11 | 25 | 18 | 4 | 93 |  |
|  |  |  |  |  |  | MST | 25 | 67 | 4 | 44 |  |
| Dong Yang | 2020 | Yang Menghao and Xiao Na | 27-Mar-24 | 114 | BMI≤18.5 kg/m2/ALB≤30 g/L | NRS-2002≥3 | 62 | 8 | 18 | 26 | Y |
|  |  |  |  |  |  | PG-SGA | 71 | 5 | 9 | 29 |  |
| Xiao-Jun Ye | 2018 | Yang Menghao and Xiao Na | 27-Mar-24 | 255 | the ESPEN diagnostic criteria | NRS-2002≥3 | 47 | 86 | 4 | 118 | Y |
|  |  |  |  |  |  | MUST | 48 | 48 | 3 | 156 |  |
|  |  |  |  |  |  | MNA-SF≤11 | 48 | 74 | 3 | 130 |  |
| Qianqian Zhang | 2021 | Yang Menghao and Xiao Na | 27-Mar-24 | 265 | ALB≤40g/L | NRS-2002≥3 | 84 | 98 | 26 | 57 | Y |
|  |  |  |  |  |  | MNA-SF≤11 | 80 | 97 | 30 | 58 |  |

### Data Extraction Table Explanation

### The data extraction table contains essential information extracted from the included studies, supporting the analysis and results of this Meta-analysis. The specific meanings of each column are as follows:

| Column Name | Meaning |
| --- | --- |
| Author | The name of the first author of the study. Identifies the primary contributor to the literature. |
| Year | The year of publication. Indicates the timeliness and context of the research. |
| Data Extractor(s) | The names of the individuals who extracted the data. Indicates who was responsible for the data extraction, ensuring transparency and traceability. |
| Date of Extraction | The date when the data was extracted. Records when the data was obtained, reflecting its currency. |
| Sample Size | The number of participants involved in the study. A crucial statistical parameter that assesses the reliability and validity of the results. |
| Reference Standard | The reference standard used in the study. Indicates the benchmarks used to validate the effectiveness of the nutritional screening tools, affecting the interpretation of the results. |
| Index Test | The nutritional screening tool or method employed. Clarifies which specific tool was evaluated, facilitating comparisons of effectiveness across different tools. |
| TP (True Positives) | The number of true positives. Refers to the number of patients correctly identified as at nutritional risk, reflecting the sensitivity of the screening tool. |
| FP (False Positives) | The number of false positives. Refers to patients incorrectly identified as at nutritional risk, impacting the specificity of the screening tool. |
| FN (False Negatives) | The number of false negatives. Refers to patients missed as being at nutritional risk, affecting the sensitivity of the screening tool. |
| TN (True Negatives) | The number of true negatives. Refers to patients correctly identified as not at nutritional risk, reflecting the specificity of the screening tool. |
| Inclusion Confirmed | Status confirming whether the study meets the inclusion criteria. Indicates the validity of the data. Y means confirmed for inclusion, and N means not included. |
