# Supplementary material for: Evaluating the accuracy of a nutritional screening tool for patients with digestive system tumors: A hierarchical Bayesian latent class meta-analysis
Source: PLoS One. 2024 Dec 20;19(12):e0316070. doi: 10.1371/journal.pone.0316070 (PMC11661584; doi:10.1371/journal.pone.0316070)
Supplement: S3 File — This file presents the results of the risk of bias and quality assessments for the included studies, conducted using standardized evaluation tools. (DOC) [file pone.0316070.s003.doc]

**Risk of Bias and Quality Assessment (Cochrane Risk of Bias Tool)**

|  | Patient Selection | | | | | Index Test | | | | Risk of Bias | | | | Flow and Timing | | | |
| --- | --- | --- | --- | --- | --- | --- | --- | --- | --- | --- | --- | --- | --- | --- | --- | --- | --- |
| study（year） | Reference Standard | | | | Applicability Concerns | Reference Standard | | | Applicability Concerns | Reference Standard | | | Applicability Concerns | Reference Standard | | | |
| Zibing Wang(2021) | Y | Y | Y | L | L | U | Y | U | L | Y | Y | L | L | Y | Y | Y | L |
| Ting Guo（2015） | Y | Y | Y | L | L | U | Y | U | L | Y | Y | L | L | Y | Y | Y | L |
| Changli Wang（2021） | Y | Y | Y | L | L | U | Y | U | H | Y | Y | L | L | Y | Y | Y | L |
| Daolai huang（2018） | Y | Y | Y | L | L | Y | U | U | L | Y | Y | L | L | Y | Y | Y | L |
| Yuqiang Liu（2017） | U | Y | Y | U | L | Y | Y | L | U | Y | Y | L | L | Y | Y | Y | L |
| Weiping Guo（2010） | Y | Y | Y | L | U | Y | Y | L | U | Y | Y | L | L | Y | Y | Y | L |
| Ping Liu（2013） | U | U | Y | U | L | Y | Y | L | L | Y | Y | L | L | Y | Y | Y | L |
| Yu Zhou（2017） | Y | Y | Y | Y | L | Y | Y | L | U | Y | Y | L | L | Y | Y | Y | L |
| Xiaojing Li（2018） | Y | Y | Y | L | L | Y | Y | L | U | Y | Y | L | L | Y | Y | Y | L |
| Wan Zhou（2015） | Y | Y | Y | L | L | Y | Y | L | L | Y | Y | L | L | Y | Y | Y | L |
| Hai Liang（2020） | Y | Y | Y | L | L | Y | Y | L | L | Y | Y | L | L | Y | Y | Y | L |
| Yage Zhu（2021） | Y | Y | Y | L | L | U | Y | U | L | Y | U | L | U | Y | Y | Y | L |
| Guibin Li（2019） | Y | Y | Y | L | U | Y | Y | L | L | Y | Y | L | L | Y | Y | Y | L |
| Juntao Chi（2017） | Y | Y | Y | L | L | U | Y | U | U | Y | Y | L | L | Y | Y | Y | L |
| Shanjun Tan（2022） | Y | Y | Y | L | L | U | Y | U | L | Y | Y | L | L | U | Y | Y | U |
| Elnaz Faramarzi（2012） | N | Y | U | H | U | Y | Y | L | U | Y | Y | L | L | Y | Y | Y | L |
| Mei-Yu Tu（2012） | Y | Y | Y | L | U | Y | Y | L | L | Y | N | H | U | Y | Y | Y | L |
| Seung Wan Ryu（2010） | U | Y | U | U | L | U | Y | U | U | Y | Y | L | L | Y | Y | Y | L |
| Bingxin Xie（2022） | Y | Y | Y | L | L | Y | Y | L | L | Y | Y | L | L | Y | Y | Y | L |
| Mariana Abe Vicente（2013） | Y | Y | Y | L | U | U | Y | U | U | Y | Y | L | L | Y | Y | Y | L |
| Taobo Jin（2010） | U | Y | Y | U | H | Y | Y | L | L | Y | Y | L | L | Y | Y | Y | L |
| Yingying Shi（2019） | Y | Y | Y | L | L | Y | Y | L | L | Y | Y | L | L | Y | Y | Y | L |
| Hong Ji（2023） | Y | Y | Y | L | L | Y | Y | L | U | Y | Y | L | L | Y | Y | Y | L |
| Li Lin（2018） | Y | Y | Y | L | L | U | Y | U | U | Y | Y | L | L | Y | Y | Y | L |
| Xi Qiao（2015） | Y | U | Y | U | U | Y | Y | L | L | Y | Y | L | L | Y | Y | Y | L |
| Bingxin Xie（2022） | Y | Y | Y | L | L | Y | Y | L | U | Y | Y | L | L | Y | Y | Y | L |
| Xite Zheng（2024） | Y | Y | Y | L | L | Y | U | U | U | Y | Y | L | U | Y | Y | Y | L |
| Shengqiang Tan（2024） | U | Y | U | U | L | Y | Y | L | L | Y | Y | L | L | Y | Y | Y | L |
| Xiaoli Ruan（2022） | U | Y | U | U | L | Y | Y | L | U | U | Y | U | L | Y | Y | Y | L |
| Reyyan Yıldırım（2020） | Y | Y | U | U | L | Y | U | U | U | Y | Y | L | L | Y | Y | Y | L |
| Dong Yang（2020） | Y | Y | Y | L | L | Y | Y | L | L | Y | U | U | Y | Y | Y | Y | L |
| Xiao-Jun Ye（2018） | Y | Y | Y | L | L | Y | Y | L | L | Y | Y | L | L | Y | Y | Y | L |
| Qianqian Zhang（2021） | Y | Y | Y | L | L | Y | Y | L | L | Y | Y | L | L | Y | Y | Y | L |

**Explanation of the QUADAS-2 Table**

The table provided uses the **QUADAS-2 (Quality Assessment of Diagnostic Accuracy Studies 2)** tool to evaluate the risk of bias and applicability concerns in the included studies. This tool is commonly used in systematic reviews and meta-analyses to assess the quality of diagnostic accuracy studies. Below, I provide a detailed explanation of the table structure and the meaning of the abbreviations used.

**Table Structure and Interpretation:**

1. **Column Headings Explanation:**

**Patient Selection**: Assesses whether the study used an appropriate method for selecting participants, such as random sampling or avoiding the exclusion of particular groups.

**Index Test**: Evaluates whether the diagnostic test under investigation was conducted and interpreted correctly, and whether the same test criteria were applied to all participants.

**Risk of Bias**: A comprehensive assessment of the study's risk of bias across all domains.

**Flow and Timing**: Examines the time interval between the administration of the index test and the reference standard, and whether all patients received the same reference standard without significant loss to follow-up.

1. **Row Headings Explanation:**

**Study (Year)**: The first author and publication year of each study included in the review.

1. **Meaning of Abbreviations in Each Column:**

**Y (Yes)**: Indicates that the study has no significant risk of bias or applicability concerns in the specified domain. It means that the study used appropriate methods for patient selection, index testing, and timing of the diagnostic process.

**N (No)**: Suggests that the study has a high risk of bias or applicability concerns in the domain. It means that there were issues such as inappropriate patient selection or inconsistent application of the index test.

**U (Unclear**): Denotes that the information provided in the study is insufficient to determine the level of risk or applicability. There may be a lack of detail in the study description, making it difficult to judge the quality of that domain.

**L (Low Risk)**: Indicates a low risk of bias in that particular domain.

**H (High Risk)**: Indicates a high risk of bias, suggesting that the study's findings may be unreliable in that domain.

1. **Detailed Explanation of Each Domain:**

**Patient Selection**:This domain assesses whether the inclusion criteria were applied consistently and without bias. A ‘Yes’ (Y) indicates that the study included participants using appropriate criteria without exclusion bias. A ‘No’ (N) or ‘High Risk’ (H) suggests potential bias in the selection of participants.

**Index Test:**Refers to the diagnostic test being evaluated. It should be applied and interpreted uniformly across all study participants. A ‘Yes’ (Y) indicates correct and consistent application, while a ‘No’ (N) or ‘High Risk’ (H) indicates variability or misapplication.

**Risk of Bias:**This is an overall judgment based on the individual domains. ‘Low Risk’ (L) suggests the study has minimal risk of bias, whereas ‘High Risk’ (H) suggests significant concerns.

**Flow and Timing:**This domain looks at whether all patients completed the study, including follow-up, and whether there was appropriate timing between the index test and the reference standard. ‘Low Risk’ (L) means the study followed all patients appropriately, while ‘High Risk’ (H) indicates potential bias due to loss to follow-up or inconsistencies in timing.

1. **Applicability Concerns:**

This category evaluates whether the study’s population, test, and reference standards are applicable to the review question. ‘Yes’ (Y) indicates no concerns, while ‘No’ (N) or ‘Unclear’ (U) suggests that the study may not be applicable to the broader clinical or research context.

**Using the Table for Interpretation:**

1. **Assessing Overall Study Quality:**

Look at the individual ratings for each study across the various domains. Studies with ‘High Risk’ (H) or ‘No’ (N) ratings in multiple domains are likely to have lower overall reliability.

1. **Identifying Key Areas of Concern:**

If a study has ‘No’ (N) or ‘High Risk’ (H) ratings in critical domains such as Patient Selection or Index Test, this indicates potential issues with how participants were selected or how the test was applied, which can impact the study’s validity.

**3.Determining Applicability:**

Use the ‘Applicability Concerns’ ratings to understand if the study’s results are generalizable to other clinical settings. If the ratings are ‘No’ (N) or ‘Unclear’ (U), the study may have limited relevance outside the specific context in which it was conducted.

**Conclusion:**

This table provides a comprehensive evaluation of the included studies using the QUADAS-2 tool. The abbreviations and ratings help to quickly identify the quality and applicability of each study, ensuring that the meta-analysis is based on reliable and relevant evidence.
