# Supplementary material for: Evaluating the accuracy of a nutritional screening tool for patients with digestive system tumors: A hierarchical Bayesian latent class meta-analysis
Source: PLoS One. 2024 Dec 20;19(12):e0316070. doi: 10.1371/journal.pone.0316070 (PMC11661584; doi:10.1371/journal.pone.0316070)
Supplement: S4 File — This file provides the Stata commands and scripts used for the hierarchical Bayesian modeling and statistical analyses performed in this study. (DOC) [file pone.0316070.s004.doc]

**Attachment: Stata Commands Used in the Analysis**

**1. Merging Statistics and Plotting HSROC Curve**

**Command Used**: *metandi*

**Purpose**: To compute summary statistics under the HSROC model and to plot the HSROC curve to evaluate the diagnostic performance of different nutritional screening tools.

**Commands**:

*metandi tp fp fn tn*

**Description**: This command calculates pooled diagnostic performance metrics, such as sensitivity and specificity.

**HSROC Curve Plotting:**

*metandi tp fp fn tn, plot nob noh*

**Description**: This command not only computes pooled statistics but also plots the HSROC curve, providing a visual representation of diagnostic performance across different screening tools.

**2. Testing for Publication Bias**

**Command Used**: *midas*

**Purpose**: To assess the impact of publication bias, particularly the small-study effect, on the research findings.

**Command:**

*midas tp fp fn tn, pubbias*

**Description**: This command performs a test for publication bias, allowing evaluation of the robustness of the study results by detecting small-study effects.

**3. Bayesian Analysis and Post-Test Probability Calculation**

**Command Used**: *midas*

**Purpose**: To calculate post-test probabilities using Bayesian analysis, which helps in understanding the clinical utility of the screening tools.

**Command**:

*midas tp fp fn tn, fagan*

**Description:** This command generates a Fagan plot to illustrate the pre-test and post-test probabilities, providing insights into the diagnostic impact of each screening tool.
