# Supplementary material for: Evaluating the accuracy of a nutritional screening tool for patients with digestive system tumors: A hierarchical Bayesian latent class meta-analysis
Source: PLoS One. 2024 Dec 20;19(12):e0316070. doi: 10.1371/journal.pone.0316070 (PMC11661584; doi:10.1371/journal.pone.0316070)
Supplement: Attachment — Submitted filename: Response to Reviewers.doc [file pone.0316070.s005.doc]

**Response to Reviewers**

**Manuscript ID**: PONE-D-24-31460

**Title**: *Evaluating the accuracy of a nutritional screening tool for patients with digestive system tumors: A hierarchical Bayesian latent class meta-analysis*

Dear Dr. Domenico Fuoco and Reviewers,

We would like to sincerely thank you for your valuable feedback and careful review of our manuscript. We greatly appreciate the opportunity to revise and resubmit our work. Below is our point-by-point response to the comments raised by the reviewers.

**Reviewer #1:**

1. **Comment**: The study’s population is localized in a specific region of China, which may limit the generalizability of the findings.

**Response**: We fully acknowledge this limitation. In the revised manuscript, we have clearly stated this in both the Abstract and Conclusion sections. We have highlighted that the findings are based on a specific Chinese population, which may affect their applicability to other regions. We have also suggested that future research should include diverse populations to further validate the effectiveness of the nutritional screening tools in broader contexts. These changes aim to provide a balanced interpretation of our results while outlining future research directions.

2. **Comment**: The statistical methods, particularly the hierarchical Bayesian analysis, require further clarification.

**Response**: We have expanded the Methods section to provide a more detailed explanation of the hierarchical Bayesian latent class meta-analysis employed in the study. This includes the rationale for selecting the hierarchical summary receiver operating characteristic (HSROC) model, which is particularly well-suited for diagnostic accuracy studies that involve multiple tools across various populations. In addition, we have included a step-by-step description of the statistical process, including the use of the *metandi* and *midas* commands in Stata to calculate pooled diagnostic performance metrics and perform Bayesian post-test probability analysis. This has been further detailed in *Supplementary Material 4*, ensuring full transparency and reproducibility of the statistical methods used.

3. **Comment**: The reviewer suggested that similar studies be conducted in different parts of the world to provide more universally applicable conclusions.

**Response**: We agree with the reviewer’s observation. In the revised Conclusion, we have explicitly discussed the need for larger, multi-regional studies to ensure the broader applicability of our findings. This is a critical step toward validating the effectiveness of nutritional screening tools across various cultural, dietary, and healthcare environments. We believe this acknowledgment will help guide future research efforts in this area.

**Reviewer’s Concern Regarding References and Data Extraction:**

1. **Comment**: Concerns were raised about the references used and the integrity of the data extraction process.

**Response**: After a comprehensive review of all references, we confirm that none of the cited studies have been retracted or are unpublished. All the references included in our manuscript are from peer-reviewed, published studies, and their status has been verified using major academic databases, including PubMed, Embase, the Cochrane Library, CNKI, Wanfang Data, and VIP.

- **Data Extraction Completeness:** The data extraction process was performed with meticulous care by two independent reviewers. All data points, including true positives (TP), false positives (FP), false negatives (FN), and true negatives (TN), were accurately extracted and cross-verified. No external or unpublished data were used in the analysis, and all extracted data are fully presented in the supplementary materials (*Supplementary Material 2*). We have also provided a comprehensive list of included and excluded studies with reasons for exclusion (*Supplementary Material 1*) to ensure complete transparency in the study selection process.
- **No Changes to Reference List**: Given that none of the references have been retracted or found erroneous, we did not make any changes to the reference list. All the cited studies are valid, published research, and we have followed appropriate academic practices in compiling our citations.

2. **Comment**: Reviewer raised concerns about the potential for bias in the data extraction process.

**Response**: To address these concerns, we employed the QUADAS-2 (Quality Assessment of Diagnostic Accuracy Studies) tool to evaluate the risk of bias and applicability for each included study. The results of this assessment are detailed in *Supplementary Material 3*. We believe this systematic evaluation mitigates any potential bias and ensures the robustness of our conclusions. In addition, we have clearly documented the Stata commands used to perform the statistical analysis in *Supplementary Material 4*, enhancing transparency and reproducibility.

**Additional Revisions:**

1. **Abstract and Conclusion**: Both sections have been revised to emphasize the study’s geographical limitation and the importance of further validation in diverse populations.
2. **Data Transparency**: A comprehensive table of the included and excluded studies, along with the reasons for exclusion, has been provided in *Supplementary Material 1*. This ensures full clarity regarding the data selection and inclusion process.
3. **Statistical Analysis Details**: Added detailed information on the statistical methods used, including the commands for generating HSROC curves and conducting Bayesian analysis, in *Supplementary Material 4*.

**General Revisions:**

1. **Financial Disclosure**: We confirm that the funders had no role in the study design, data collection, data analysis, decision to publish, or preparation of the manuscript. This has been clearly stated in the revised Cover Letter.
2. **Supporting Information**: The headings of the supplementary material have been updated and in-text citations have been reformatted to conform to journal guidelines..
3. **Figure Adjustments**：We have revised the figures to adhere to PLOS ONE formatting guidelines. Specifically:We ensured that all figures adhere to the journal’s specifications regarding dimensions, font size, and figure legends. These adjustments ensure that the figures are presented in a clear, professional manner, meeting the expectations of both the reviewers and the journal’s editorial policies.

**Additional Response to Editorial Request (Regarding Data Availability Statement):**

1. **Comment**: You requested clarification and confirmation regarding the data availability plan, highlighting the importance of having a clear, actionable plan in place before acceptance. You mentioned that the entire dataset must be made freely accessible upon publication, unless there are ethical or protocol compliance concerns.

**Response:** We have revised the Data Availability Statement as requested. The dataset has been made available through the Dryad repository (DOI: doi:10.5061/dryad.4mw6m90m8), which includes all relevant extracted data points used in the meta-analysis (true positives, false positives, false negatives, true negatives), as well as the Stata commands employed for statistical analysis. We have ensured that the data have been anonymized and are in full compliance with ethical guidelines.

**Attachments Included:**

1. **Revised Manuscript with Tracked Changes**: Labeled as 'Revised Manuscript with Track Changes.'
2. **Clean Revised Manuscript**: Labeled as 'Manuscript.'
3. **Revised Cover Letter**: Highlighting changes made based on the reviewers’ comments and updating the role of funders statement.
4. **Response to Reviewers**: This document, detailing responses to each comment and the revisions made.
5. **Supplementary Materials**: Including data extraction, risk of bias assessment, and statistical methods (refer to *Supplementary Materials 1, Supplementary Materials 2, Supplementary Materials 3*, and *Supplementary Materials 4*).

We are confident that these revisions address the reviewers' concerns and enhance the overall quality and rigor of the manuscript. We thank the reviewers and editorial team once again for the opportunity to improve our work and look forward to your feedback.

Yours sincerely,

Xiuqin Yang

Department of General Surgery

The Second Affiliated Hospital of Guizhou Medical University

Kaili City, Qiandongnan Prefecture, Guizhou Province, China

Email: 3129863401@qq.com

Phone: +86 18888107735
